# Association of polymorphisms in genes of factors involved in regulation of splicing of cystic fibrosis transmembrane conductance regulator mRNA with acute respiratory distress syndrome in children with pneumonia

**DOI:** 10.1186/s13054-016-1454-7

**Published:** 2016-09-05

**Authors:** Francesca Perez-Marques, Pippa Simpson, Ke Yan, Michael W. Quasney, Nadine Halligan, Daniel Merchant, Mary K. Dahmer

**Affiliations:** 1 Section of Critical Care Medicine, Medical College of Wisconsin, Milwaukee, WI USA; 2 Section of Quantitative Health Sciences, Department of Pediatrics, Medical College of Wisconsin, Milwaukee, WI USA; 3 Children’s Research Institute, Medical College of Wisconsin, Milwaukee, WI USA; 4 Human and Molecular Genetics Center, Medical College of Wisconsin, Milwaukee, WI USA; 5 Division of Pediatric Critical Care, Department of Pediatrics and Communicable Diseases, University of Michigan, 1500 East Medical Center Dr, SPC 5243, Ann Arbor, MI 48109-5243 USA

**Keywords:** ARDS, ALI, Acute lung injury, Pediatrics, Genetic association study, Pneumonia

## Abstract

**Background:**

Previous work has demonstrated a strong association between lung injury in African American children with pneumonia and a polymorphic (TG)_m_T_n_ region in cystic fibrosis transmembrane conductance (CFTR) involved in the generation of a nonfunctional CFTR protein lacking exon 9. A number of splicing factors that regulate the inclusion/exclusion of exon 9 have been identified. The objective of this study was to determine whether genetic variants in these splicing factors were associated with acute respiratory distress syndrome (ARDS) in children with pneumonia.

**Methods:**

This is a prospective cohort genetic association study of lung injury in African American and non-Hispanic Caucasian children with community-acquired pneumonia evaluated in the emergency department or admitted to the hospital. Linkage-disequilibrium-tag single nucleotide polymorphisms (LD-tag SNPs) in genes of the following splicing factors (followed by gene name) involved in exon 9 skipping PTB1 (*PTBP1*), SRp40 (*SFRS1*), SR2/ASF (*SFRS5*), TDP-43 (*TARDBP*), TIA-1 (*TIA1*), and U2AF^65^ (*U2AF2*) were genotyped. SNPs in the gene of the splicing factor CELF2 (*CELF2*) were selected by conservation score. Multivariable analysis was used to examine association between genotypes and ARDS.

**Results:**

The African American cohort (*n* = 474) had 29 children with ARDS and the non-Hispanic Caucasian cohort (*n* = 304) had 32 children with ARDS. In the African American group multivariable analysis indicated that three variants in *CELF2*, rs7068124 (*p* = 0.004), rs3814634 (*p* = 0.032) and rs10905928 (*p* = 0.044), and two in *TIA1*, rs2592178 (*p* = 0.005) and rs13402990 (*p* = 0.018) were independently associated with ARDS. In the non-Hispanic Caucasian group, a single variant in *CELF2*, rs2277212 (*p* = 0.014), was associated with increased risk of developing ARDS.

**Conclusions:**

The data indicate that SNPs in *CELF2* may be associated with the risk of developing ARDS in both African American and non-Hispanic Caucasian children with pneumonia and suggest that the potential role of the splicing factor CELF2 in ARDS should be explored further.

**Electronic supplementary material:**

The online version of this article (doi:10.1186/s13054-016-1454-7) contains supplementary material, which is available to authorized users.

## Background

Community-acquired pneumonia (CAP) is an important worldwide cause of pediatric morbidity and mortality. The annual incidence of CAP in European and North American children less than 5 years old is higher (34 to 40 cases per 1000) than the incidence seen at any other time of life, with the exception of the elderly (>75–80 years) [1, 2]. In children in the USA, pneumonia accounts for almost a third of all respiratory-related hospitalizations [3] with the burden of hospitalization highest for children under 5 years of age [4]. In the developing world the incidence of pneumonia in children under 5 is reported to be 6 to 10 fold higher than that of developed countries and mortality is also higher with a reported rate of 1.3–2.6 % [5–10].

The response to pneumonia varies between individuals. Some individuals with pneumonia have relatively mild disease while others go on to develop acute respiratory distress syndrome (ARDS). The underlying pathophysiological processes observed in ARDS include disruption of the alveolar capillary barrier, alveolar edema, inflammation and, in some cases, fibrosis [11, 12]. Studies suggest that pediatric ARDS (PARDS) is similar to ARDS in adults though the immaturity of the lung parenchyma and immune systems in children suggest that there may be significant differences in the development and outcome of ARDS in children [13]. A number of variants in genes for proteins involved in the pathophysiology of ARDS have been reported to be associated with development of ARDS or outcome from ARDS [14–16], though most of the studies have been performed in adults. These observations suggest that genetic variability present in genes that encode for proteins involved in ARDS pathophysiology may play an important role in the variability observed in the severity of CAP-induced lung injury.

Several studies in children have also identified the association between genetic variants and lung injury or PARDS [13]. Recently we demonstrated a strong association in African American children with pneumonia between lung injury and a polymorphic (TG)_m_T_n_ site located close to the splice junction between intron 8 and exon 9 of the cystic fibrosis transmembrane conductance regulator (CFTR) gene [17]. The (TG)_m_T_n_ site is linked to a defect in CFTR messenger RNA (mRNA) splicing and reduced functional CFTR protein [18–24]. The observed association with ARDS supports other findings indicating that CFTR, a chloride channel found in epithelial cells [25] and polymorphonuclear blood cells [26–29], has a role in the development of ARDS. CFTR is important in fluid clearance in the injured lung [30, 31], is a negative regulator of the inflammatory response [32–34], and the lack of functional CFTR on neutrophils enhances development of ARDS triggered by lipopolysaccharide (LPS) derived from gram negative bacteria in a mouse model [28]. Together these results indicate that CFTR plays a role in ARDS in children with pneumonia and predict that genetic variants that affect the expression of functional CFTR, including variants in proteins involved in the regulation of CFTR mRNA splicing, will be associated with ARDS.

The (TG)_m_T_n_ region in *CFTR* is composed of variable numbers of TG and T repeats and affects the inclusion/exclusion of exon 9. Alleles with 12 or more TGs and/or 5 or fewer Ts are associated with skipping of exon 9 and synthesis of a non-functional CFTR protein missing part of the nucleotide-binding domain-1 [18–24]. Regulation of inclusion/exclusion of exon 9 during mRNA maturation has been well-studied and a number of proteins involved in splicing, and regulation of splicing, in that region have been identified. Such proteins include CUGBP, elav-like family member 2 (CELF2), HIV-1 TAR DNA binding protein (TDP-43), polypyrimidine tract binding protein (PTB), two SR proteins (SF2/ASF, SRp40), T-cell intracellular antigen 1 (TIA-1), and U2AF^65^ [35–38]. Based on our earlier study showing association between specific alleles of (TG)_m_T_n_ and acute lung injury [17] we hypothesized that polymorphisms in genes of the splicing factors involved in regulation of exon 9 skipping would be associated with ARDS in children with pneumonia.

## Methods

### Study design

We performed a genetic association study to examine if polymorphisms in splicing factors involved in exon 9 splicing of the CFTR gene are associated with ARDS in pediatric patients enrolled in a prospective, multicenter study of the role of genetic variation in lung injury in children with community acquired pneumonia (CAP). The outcome measure was a PaO_2_/FiO_2_ ratio ≤300, bilateral infiltrates, and the absence of evidence of left atrial hypertension which at the time the study was initiated was defined as acute lung injury by the American European Consensus Committee [39]. However, as the more recent definitions of ARDS in both adults [40] and children [41] have discontinued the use of the term acute lung injury, and patients with a ratio of partial pressure of oxygen (PaO2)/inspired oxygen fraction (FiO_2_) ≤300 are currently considered to have ARDS, for the purposes of this report we will refer to all patients with a PaO_2_/FiO_2_ ratio ≤300 as having ARDS.

### Study population

Children (2 weeks to 18 years old) of any race or ethnicity presenting to the Emergency Room, inpatient unit or pediatric intensive care unit with a diagnosis of CAP at either Le Bonheur Children’s Hospital, Children’s Memorial Hospital, or Children’s Hospital of Wisconsin were eligible for the study. The Institutional Review Board (IRB) from each institution (University of Tennessee Health Science Center IRB, Children’s Memorial Hospital IRB, Children’s Hospital of Wisconsin IRB) approved the study. Consent was obtained by individuals or their guardians for all participants except for patients enrolled from the emergency department at Children’s Hospital of Wisconsin where the IRB granted a waiver of consent and for patients from the emergency department at Le Bonheur Children’s where the IRB waived consent if parents or guardians could not be contacted.

Analysis was stratified by race, as the frequency of genetic polymorphisms and the linkage disequilibrium patterns differ between ethnicities and races [42, 43], and such groups should be analyzed separately [44]. Only African Americans and non-Hispanic Caucasians (52 % and 35 % of the cohort, respectively) were examined, as only these two groups had a sufficient number of individuals for meaningful analysis. Race was self-reported. This cohort was used previously to examine the association between (TG)_m_T_n_ alleles and acute lung injury [17] and some members of this cohort have been used in earlier studies [45, 46].

The inclusion criteria were children (2 weeks to 18 years old) with CAP defined as: 1) acute illness (<14 days of symptoms); 2) new infiltrate on chest x-ray; 3) clinical features compatible with pneumonia, including one of the following: fever >37.8 °C, hypothermia <36 °C, peripheral white blood count >10,000/μL or <4500/μL, or >15 % immature neutrophils; and two of the following: tachypnea (respiratory rate >2 standard deviations from the mean for age), dyspnea, or hypoxemia (pulse oximetry ≤94 % on room air on initial evaluation without a known right to left cardiac shunt). Exclusion criteria were diagnosis of immunodeficiency (primary or acquired, including treatment with immunosuppressant drugs), congenital heart disease, malignancy or history of malignancy, need for oxygen home therapy, cystic fibrosis or residence at a chronic care facility within the previous 30 days or hospitalization within the previous 7 days. For the purposes of this study we have only examined African American and non-Hispanic Caucasian children.

### Selection of single nucleotide polymorphisms for genotyping

The splicing factor genes selected were identified as potential candidate genes related to ARDS after demonstration that the *CFTR* (TG)_m_T_n_ polymorphic site involved in regulation of exon 9 skipping was associated with acute lung injury [17]. The splicing factors (followed by their gene name) specifically involved in regulating exon 9 skipping in the CFTR gene [35–38] include: CELF2 (*CELF2*), PTB (*PTBP1*), SRp40 (*SFRS1*), SR2/ASF (*SFRS5*), TIA-1 (*TIA1*), TDP-43 (*TARDBP*), and U2AF^65^ (*U2AF2*). Linkage disequilibrium-tag single nucleotide polymorphisms (LD-tag SNPs) were identified in *PTBP1*, *SFRS1*, *SFRS5*, *TARDBP*, *TIA1*, and *U2AF2* using the program LD-select (available on the Seattle SNPs website http://gvs.gs.washington.edu/GVS/) which selects an optimal set of LD-tag SNPs based on the *r*^2^ LD statistic [47]. The program was run with an *r*^2^ threshold of 0.8 using the HAPMAP-CEU or –YRI SNP genotyping database or the African American SNP genotyping data from the Programs for Genomic Applications database.

SNP selection included the region extending from 5 kb upstream of the transcription start site to 2 kb downstream of the coding region to cover 5′ or 3′ regions involved in regulating gene expression. The minor allele frequency cutoff was 3 %. In African Americans 7 LD-tag SNPs in *PTBP1*, 5 in *SFRS1*, 5 in *SFRS5*, 11 in *TARDBP*, 12 in *TIA1*, and 12 in *U2AF2* were identified. In Caucasians, 6 LD-tag SNPs in *PTBP1*, 3 in *SFRS1*, 4 in *SFRS5*, 5 in *TARDBP*, 3 in *TIA1*, and 10 in *U2AF2* were identified. There were five LD-tag SNPs that could not be genotyped successfully due to failure in the design, manufacturing, or genotyping assay including rs9430171 in *TARDBP*, and rs504850 and rs2271757 in *U2AF2* in both African Americans and Caucasians, rs8100561 (*PTBP1*) in African Americans only, and rs188701 (*U2AF2*) in Caucasians only.

A different strategy was used for *CELF2*, as 331 and 162 LD-tag SNPs were identified in the cohorts of African and European descent, respectively. Consequently, SNPs genotyped in the *CELF2* gene were selected by conservation score using PhastCons [48] and GERP [49]. A PhastCons score >0.7 and a GERP score >2 were used as selection criteria. A total of 20 or 15 SNPs for the *CELF2* gene were identified in individuals of African and European descent, respectively. One of the SNPs in the African American cohort failed the custom design process (rs201112) and the Taqman assay for rs790441 failed in both groups.

*A priori* power calculations using Quanto 1.2.3 [50, 51] indicated that the African American and non-Hispanic Caucasian cohorts were powered (using 80 % power) to observe associations between SNPs and ARDS (using population estimates for minor allele frequency from the HAPMAP or Programs for Genomic Applications database), giving minimal detectable odds ratios of approximately 2.1 to 4.3 depending upon the minor allele frequency. SNPs with minor allele frequencies between 10 % and 49 % (77 % and 66 % of the selected SNPs in the African American and non-Hispanic Caucasian groups, respectively) had minimal detectable odds ratios of 2.1 to 2.7, while those for frequencies between 3 % and 10 % ranged from 4.3 to 2.7.

### Genotyping

DNA was extracted from whole blood using the Wizard Genomic DNA Purification Kit (Promega, Madison, WI, USA). Genotyping was performed blinded to the clinical status of individuals. Genotyping of the LD-tag SNPs was performed using 5′ exonuclease TaqMan assays (Applied Biosystems, Foster City, CA, USA).

For all genotyping assays approximately 5–10 % of randomly chosen samples were genotyped a second time to verify reproducibility; average concordance was 99.9 % (104 assays had 100 % concordance and 3 others had 96.4–97.8 % concordance). Call rates ranged from 99.4–100 %. Genotype frequencies of sites did not deviate significantly from Hardy-Weinberg equilibrium (significance set at *p* < 0.01) except for rs3765896 (*TARDBP*) in the African American cohort.

### Statistical analysis

For the univariable analysis, either the two-sided chi-square test or Fisher’s Exact test (when any cell in the cross-tabulation had counts less than 5) was performed to test for association between categorical variables and the presence of ARDS. The Mann–Whitney *U* test was used for the continuous variables (age, hospital length of stay, room air pulse oximetry). Predictors that were tested besides age included the various alleles in the splicing genes (grouped as the presence of one or two copies of specific alleles), the (TG)_m_T_n_ site in CFTR (grouped as 1 or 2 copies of high risk alleles), gender, asthma, history of bronchopulmonary dysplasia, neurologic disorder (defined as developmental delay and/or history of seizures), and sickle cell anemia. As there was a gap in age in this group, age was also categorized into two groups for the analysis, <11 years and ≥11 years. SNPs with a *p* value <0.2 were included in the multivariable analysis along with the covariates.

Multivariable logistical regression analysis was performed using the development of ARDS as the dependent variable. Initially variables, such as SNPs, were selected for consideration in the model if the *p* value was <0.2 in the univariate analysis. A stepwise selection approach, with 0.05 and 0.1 as significance levels for entry into and staying in the model, was used for selection of the variables in the final model. The exception was presence of asthma, which was always included. SAS 9.2 (SAS Institute Inc., Cary, NC, USA) was used for all the analyses.

## Results

The demographic characteristics of the African American and non-Hispanic Caucasian cohorts used for this study have been described previously [17]. The African American cohort is composed of 474 children (53 % male) 14 days to 18 years old with a median age of 2.1 years. Comorbid conditions include asthma (20 %), sickle cell disease (12 %), neurological disorders (8 %) defined as seizures and/or developmental delay, and history of bronchopulmonary dysplasia (2 %). Of these patients 43 were mechanically ventilated, 29 had ARDS and 3 died. The non-Hispanic Caucasian group includes 304 children (54 % male) 18 days to 17 years old with a median age of 4.0 years. Among these patients, 15 % have asthma, 8 % have neurological disorders and 2 % have a history of bronchopulmonary dysplasia, and 40 patients were mechanically ventilated, 32 had ARDS and 2 died.

Shown in Table 1 is a comparison of general characteristics between children with and without ARDS in these cohorts. The demographic characteristics and co-morbidities do not differ significantly between children with or without ARDS in either the African American or non-Hispanic Caucasian children except that in the non-Hispanic Caucasian cohort the ARDS group is older (Table 1). As expected clinically related measures of disease are significantly different between those with and without ARDS. Of the African American children with admission measurements of room air pulse oximetry (*n* = 20 patients with ARDS and 397 patients without ARDS) saturation in room air was 87 % (range 50–98) and 95 % (range 48–100), respectively (*p* < 0.001); for non-Hispanic Caucasian children with measurements available (*n* = 23 patients with ARDS and 250 patients without ARDS) saturation was 89 % (range 50–99) and 93 % (range 55–100), respectively (*p* = 0.001). Approximately 85 % of the patients with ARDS in each cohort had a PaO_2_/FiO_2_ ≤ 200.

**Table 1:** Comparison of general characteristics of patients with and without acute respiratory distress syndrome

Characteristics	No ARDS	ARDS	*p* ^a^
African Americans			
Age (years), median (range)	2.1 (14 days to 18.9)	5.9 (25 days to 17.9)	0.19
Gender, *n* (%) male	230 (52)	19 (66)	0.15
Comorbidities, *n* (%)			
Asthma	94 (21)	3 (10)	0.16
Bronchopulmonary dysplasia^b^	9 (2)	1 (3)	0.47
Neurological disorders^c^	31 (7)	5 (17)	0.06
Sickle cell disease	54 (12)	3 (10)	>0.99
Mortality, *n* (%)	0 (0)	3 (10)	<0.001
Admitted to hospital, *n* (%)	274 (62)	29 (100)	<0.001
Admitted to PICU, *n* (%)	15 (3)	29 (100)	<0.001
Mechanical ventilation, n (%)	14 (3)	29 (100)	<0.001
Hospital length of stay^d^, median (range)	2 (0–19)	22 (6–101)	<0.001
PaO_2_/FiO_2_, *n* (%)			
≤ 100	na	16 (55)	-
101 – ≤ 200	na	8 (28)	-
201 – ≤ 300	na	5 (17)	-
Non-Hispanic Caucasians			
Age (years), median (range)	3.7 (18 days to 17.8)	9.6 (63 days to 17.9)	<0.001
Gender, *n* (%) male	151 (56)	14 (44)	0.21
Comorbidities, *n* (%)			
Asthma	44 (16)	2 (6)	0.19
Bronchopulmonary dysplasia^b^	6 (2)	0 (0)	>0.99
Neurological disorders^c^	19 (7)	5 (16)	0.15
Mortality, *n* (%)	0 (0)	2 (6)	0.01
Admitted to hospital, *n* (%)	209 (77)	32 (100)	<0.001
Admitted to PICU, *n* (%)	17 (6)	32 (100)	<0.001
Mechanical ventilation, *n* (%)	12 (4)	30 (94)	<0.001
Hospital length of stay^d^, median (range)	3 (0–69)	17 (6–126)	<0.001
PaO_2_/FiO_2_, *n* (%)			
≤ 100	na	14 (44)	-
101 – ≤ 200	na	14 (44)	-
201 – ≤ 300	na	4 (12)	-

^a^ *p* value determined by Mann–Whitney test (age, hospital length of stay) or chi-square test or Fisher’s Exact test (others); ^b^history of bronchopulmonary dysplasia (no subjects were on oxygen therapy at home); ^c^neurological disorders included seizures and/or developmental delay; ^d^hospital length of stay in days was calculated for survivors. *n* = 474 for African Americans and 304 for non-Hispanic Caucasians. *ARDS* acute respiratory distress syndrome, *PICU* pediatric intensive care unit, *PaO*_*2*_ */FiO*_*2*_ partial pressure of oxygen/inspired oxygen fraction, *na* not available

Polymorphic sites in genes for splicing proteins involved in the regulation of inclusion or exclusion of exon 9 in CFTR were genotyped. The SNPs selected for genotyping were either LD-tag SNPs (*PTBP1*, *SFRS1*, *SFRS5, TIA1*, *TARDBP*, *U2AF2*) or were highly conserved sites (*CELF2*) as described in “Methods”. The minor allele, and the minor allele frequency, of the SNPs genotyped in African American and non-Hispanic Caucasian children with CAP are shown in Table S1 in Additional file 1. The minor allele frequencies are similar to those reported previously.

Multivariable logistic regression analysis was performed to determine whether any of the genetic variants in the splicing factors were associated with development of ARDS after the genotype at the (TG)_m_T_n_ site in *CFTR* and other demographic or clinical factors that may influence the severity of disease were considered. Variables included in the analysis were age, gender, comorbid conditions, presence of one or more high risk (TG)_m_T_n_ allele, and any SNPs with a *p* value <0.2 in the univariate analysis (Table 2). In African American children, multivariable analysis indicated that three SNPs in the *CELF2* gene (rs7068124, rs3814634, rs10905928; odds ratio (OR) = 4.28, 2.95, and 2.66, respectively) and two SNPs in the *TIA1* gene (rs25921789, rs13402990; OR = 3.70 and 5.42, respectively) were significantly associated with development of ARDS (Table 3). The high risk (TG)_m_T_n_ alleles previously reported to be associated with ARDS [17] remained independently associated with ARDS (OR = 3.01) even in the presence of the indicated splicing variants. In Caucasians a variant in *CELF2*, rs2277212 (OR = 3.22), was associated with increased risk of development of ARDS.

**Table 2:** Splicing factor variants included in the multivariable analysis

	African Americans		Non-Hispanic Caucasians	
GENE	SNPs	*p*	SNPs	*p*
*PTBP1*	rs351974	0.14		
*SFRS1*	rs2233905	0.10		
	rs2233906	0.15		
*TARDBP*	rs3765896	0.11	rs3765896	0.13
	rs1782455	0.12		
*TIA1*	rs2289920	0.16	rs2592177	0.04
	rs2166451	0.16		
	rs2592178	0.05		
	rs13402990	0.06		
	rs13024392	0.06		
	rs2921711	0.11		
*U2AF2*	rs7247677	0.14	rs7247677	0.05
	rs537728	0.09	rs617073	0.04
	rs310445	0.12	rs10420401	0.11
*CELF2*	rs3814634	0.09	rs3814634	0.08
	rs10905928	0.12	rs10905928	0.10
	rs2209285	0.15	rs2277212	0.02
	rs17149511	0.19		
	rs7068124	0.01		

*p* value determined with the chi-square or Fisher’s exact test. *SNP* single nucleotide polymorphism

**Table 3:** Multivariable analysis of association between splicing factors variants and acute respiratory distress syndrome

Variable	Odds ratio	95 % Confidence interval	*p*
African Americans			
*CELF2:* rs3814634 (GT/GG vs TT)	2.95	1.10–8.06	0.032
*CELF2:* rs7068124 (CT/TT vs CC)	4.28	1.58–11.64	0.004
*CELF2:* rs10905928 (AC/AA vs CC)	2.66	1.03–6.89	0.044
*TIA1:* rs2592178 (AG/AA vs GG)	3.70	1.49–9.15	0.005
*TIA1:* rs13402990 (AT vs AA)	5.42	1.34–21.9	0.018
High risk (TG)_m_T_n_ alleles^a^	3.01	1.27–7.14	0.012
Age group (≥11 years)	14.9	5.53–40.1	<0.0001
Asthma	0.55	0.15–2.00	0.36
Non-Hispanic Caucasians			
*CELF2*: rs2277212 (TT vs AA/AT)	3.22	1.26–8.33	0.014
Age group (≥11 years)	9.20	3.93–21.6	<0.0001
Asthma	0.20	0.04–0.96	0.04

^a^Children with one or more high risk (TG)_m_T_n_ allele compared to children with no high risk (TG)_m_T_n_ alleles; *n* = 463 for African Americans, *n* = 304 for Non-Hispanic Caucasians

As the SNPs examined in the *CELF2* gene are not LD-tag SNPs but rather were chosen by conservation scores we examined whether the three SNPs in the African American cohort associated with increased risk for ARDS are in significant linkage disequilibrium. As shown in Table 4, the three SNPs in *CELF2* do not have a high degree of linkage disequilibrium. The degree of linkage disequilibrium between the two SNPS in *TIA1* that were associated with ARDS was also very low.

**Table 4:** Linkage disequilibrium of variants associated with acute respiratory distress syndrome in individuals of African Descent

Gene	SNP1	SNP2	r^2^ ^a^
*CELF2*	rs3814634	rs7068124	0.055
	rs3814634	rs10905928	0.001
	rs7068124	rs10905928	0.018
*TIA1*	rs2592178	rs13402990	0.004

^a^r^2^ statistic from HAPMAP data for Yoruban cohort

## Discussion

This study demonstrates that there is a significant association between specific variants in *CELF2,* a gene encoding for a splicing factor involved in controlling the level of exon 9 skipping in CFTR, and risk of developing ARDS in children with pneumonia who do not suffer from cystic fibrosis. Multivariable analysis indicated that in African American children three variants in *CELF2*, rs7068124, rs3814634 and rs10905928, were independently associated with ARDS. In addition, the previously reported association between high risk (TG)_m_T_n_ alleles and ARDS remained independently associated with ARDS even when variants in genes encoding for splicing factors were included in the analysis.

Our previous study showed association between high-risk (TG)_m_T_n_ alleles in the CFTR gene and ARDS in African Americans but not in non-Hispanic Caucasians. One possible explanation for this difference is that intracellular levels of splicing factors differ between Caucasians and African Americans resulting in a difference in the degree of exon 9 skipping and consequently the level of functional CFTR with identical (TG)_m_T_n_ alleles. As in vitro data indicate that modulating the amount of TDP-43 affects the amount of exon 9 skipping with a given (TG)_m_T_n_ site [35], we hypothesized that genetic variants that were associated with differences in levels of splicing factors might be associated with ARDS even in the absence of association with high-risk (TG)_m_T_n_ alleles. A genetic variant in *CELF2* (rs2277212) was associated with risk of ARDS in non-Hispanic Caucasian children. Given that genetic structure and linkage-disequilibrium varies significantly between African Americans and non-Hispanic Caucasians, it is not surprising that the *CELF2* variant associated with the risk of ARDS in non-Hispanic Caucasian children is not one of the three identified in the African American cohort.

The *CELF2* SNPs associated with ARDS do not change the amino acid sequence of CELF2, but appear to be in transcriptional regulatory regions when evaluated using HaploReg [52]. Epigenetic modifications on histones demonstrate that rs2277212 is in an enhancer region in B lymphocytes, primary monocytes and T cells. Interestingly, this variant is in a binding motif for the transcription factor STAT. The SNP at site rs7068124 is also in a region with epigenetic marks on histones indicative of an enhancer region in primary T helper cells, and in individuals of African descent it is in linkage disequilibrium with an SNP that alters a number of DNA binding protein motifs. The variants rs10905928 and rs3814634 are in regions that have been predicted to bind a number of proteins that regulate transcription. In addition, the ENCODE database indicates that rs10905928 is in a region with a high level of monomethylated H4K20, an epigenetic modification that has been implicated in a number of processes including transcriptional regulation and DNA replication [53, 54]. The location of these variants in regulatory regions suggest that they may be involved in regulating CELF2 protein levels; however, future studies will be required to determine whether these variants are associated with regulation of CELF2 levels and whether they are indeed the causative sites.

The three sites in *CELF2* associated with ARDS in African Americans are not in significant LD suggesting that there may be multiple sites in the *CELF2* gene that have a functional impact on CELF2 in this population. Consequently, in future studies each of these three *CELF2* SNPs, and all of the SNPs with which they are in significant LD, will need to be examined for possible effects on expression of *CELF2* and binding of their putative binding protein regulatory proteins. Such studies will also be required for the *CELF2* SNP associated with ARDS in the Caucasian population.

In African American children, two variants in *TIA1,* rs2592178 and rs13402990, were also associated with risk of developing ARDS. Epigenetic modifications in the region surrounding rs2592178 suggest it is located in a promoter and/or enhancer region in a number of cell types including primary monocytes, T cells and neutrophils. The potential role of the variant rs2592178 on expression of *TIA1* is also indicated by data identifying it as an expression quantitative trait locus [52]. rs13402990 is not in a regulatory region itself but it is in complete LD with five other SNPs in individuals of African descent that are in the promoter and/or an enhancer region in many cell types including monocytes, T cells, and neutrophils, and some of these SNPs alter regulatory binding protein motifs. There was no association between genetic variants in *TIA1* and ARDS in non-Hispanic Caucasians. Interestingly, the two polymorphic sites associated with ARDS in African Americans are not polymorphic in non-Hispanic Caucasians. It is unclear whether the lack of replication between these two groups is due to a significant difference between African Americans and Caucasians or whether the association observed in the African Americans is a false positive. Future studies will have to resolve this question.

This is the first report that variations in genes that regulate mRNA splicing are associated with development of ARDS. The specific variants associated with ARDS in *CELF2* and *TIA1* have not been reported to be associated with other diseases. However, there are reports that other very rare variants in *CELF2* [55–57] and *TIA1* [58] are associated with other disease states. The variants associated with ARDS in this study do not result in a change in the amino acid sequence of the protein. However, these variants appear to be in regulatory regions of their respective gene, consequently, they may be associated with differences in levels of their protein product. The *CELF2* gene is also in a region in which structural genetic variants have been reported (http://www.ncbi.nlm.nih.gov/dbvar/?term=celf2), so it is possible that the SNPs associated with ARDS are in linkage disequilibrium with regions of the gene with deletions or insertions.

Because the genes examined in this study were chosen based on their biological role in the regulation of exon 9 skipping in *CFTR*, it is tempting to assume that the association between these SNPs and ARDS is due to their effect on splicing factors affecting the splicing of CFTR. However, the proteins encoded by *CELF2* and *TIA1* are involved in regulating the splicing of many different genes and can also affect translation, mRNA stability, and in the case of TIA-1, transcription [59–61]. Interestingly, *CELF2* expression is increased during T-cell signaling, resulting in widespread changes in splicing and in the T-cell transcriptome [62]. Future studies will be needed to determine whether the increased risk of development of ARDS observed with these variants is also seen in other cohorts and whether association between these variants and risk of ARDS is due solely, or even in part, to their impact on the amount of functional CFTR. The association between a variant in *CELF2* and decreased risk of ARDS without any association between the high risk (TG)_m_T_n_ alleles and ARDS in non-Hispanic Caucasians suggests that CELF2 may impact the risk of developing ARDS by other means in addition to the effect of exon 9 inclusion/exclusion in CFTR.

The observation that two splicing genes involved in exon 9 skipping in *CFTR* contain variants that appear to impact upon the risk of ARDS in African American children suggests that the previously observed association between (TG)_m_T_n_ and ARDS in African American children with CAP may be meaningful. In addition, the finding that genetic variants in *CELF2*, *TIA1* and the (TG)_m_T_n_ site in *CFTR* are all independently associated with ARDS suggests that children with multiple variants are likely to be at greater risk of developing CAP-induced ARDS than children with fewer variants. A larger cohort of African American children with ARDS will be required to examine this question.

There are several limitations to this study. The relatively small size of the cohort resulted in the study being powered to identify associations with minimum odds ratios of 2.1–2.7 for SNPs with minor allele frequencies between 0.49 and 0.1, and 2.7–4.2 for minor allele frequencies between 0.09 and 0.03. Consequently, there was limited power for SNPs with minor allele frequencies below 0.10 and variants with smaller effects may not have shown a statistically significant association with ARDS. Another limitation is that there are not yet any data demonstrating that the variants associated with ARDS have a functional impact on the level or function of the corresponding protein. However, the *CELF2* variants are at highly conserved sites and in regions that appear to be involved in transcriptional regulation, suggesting that they may be associated with the level of CELF2. The children that were identified as having ARDS in this study had a PaO_2_/FiO_2_ ratio ≤300 and bilateral chest infiltrates, which at the time of study design and enrollment were components of the definition of acute lung injury published by the American European Consensus Committee [39]. However, the newly recommended definitions of ARDS in both adults [40] and children [41] expanded the respiratory criterion for ARDS to include patients who previously met the criterion for either acute lung injury or ARDS and have recommended discontinuing the use of the term acute lung injury. Although the new definition of pediatric ARDS recommends using the oxygenation index (OI) or oxygen saturation index (OSI) as the respiratory criterion for ARDS, a PaO_2_/FiO_2_ ratio ≤300 was used in our cohorts because OI or OSI could not be calculated for all the patients in our cohort. Last, most of the patients with ARDS in this study had moderate to severe ARDS, so the findings might not be generalizable to patients with mild ARDS.

## Conclusions

In summary, multivariable analysis adjusted for the (TG)_m_T_n_ site in *CFTR* and for demographic and clinical factors, has identified an association between polymorphisms in *CELF2* and risk of developing ARDS in both African American and non-Hispanic Caucasian children with pneumonia. This is the first study to report association between variants in proteins involved with regulation of mRNA splicing and stability with ARDS. This finding supports the previously observed association between high risk (TG)_m_T_n_ alleles in *CFTR* and lung injury in African American children with pneumonia and suggests that CFTR and splicing factors that affect levels of functional CFTR may be involved in the degree of lung injury observed in African American and potentially in non-Hispanic Caucasian children with pneumonia. However, future studies will be required to determine whether this association can be replicated in additional, larger cohorts, whether these SNPs are associated with altered levels of protein product, and whether their effect is mediated by an effect on CFTR mRNA splicing. Such studies will begin to answer the question of whether these genetic variants should be considered in studies examining whether biomarkers are able to help predict those at greatest risk of developing ARDS and whether agents that modify CFTR function might be therapeutically useful in a subset of patients at risk of developing ARDS.

## Key messages

- Genetic variants in *CELF2*, a gene encoding a protein involved in regulation of splicing of CFTR mRNA, are associated with ARDS in both African American and non-Hispanic Caucasian children with pneumonia
- *CELF2* variants associated with ARDS are in regions involved in transcriptional regulation

## Additional file

Additional file 1: Table S1. Table of minor alleles and minor allele frequencies of genotyped variants. A table of the minor alleles and minor allele frequencies of genotyped variants in African American and Non-Hispanic Caucasian children with community acquired pneumonia. (DOCX 39 kb)

## Abbreviations

ARDS: Acute respiratory distress syndrome
CAP: Community acquired pneumonia
CELF2: CUGBP, elav-like family member 2
CFTR: Cystic fibrosis transmembrane conductance regulator
FiO_2_: Inspired oxygen fraction
IRB: Institutional Review Board
OI: Oxygenation index
LD-tag SNPs: Linkage-disequilibrium-tag single nucleotide polymorphisms
LPS: Lipopolysaccharide
mRNA: messenger RNA
OSI: Oxygen saturation index
PARDS: Pediatric acute respiratory distress syndrome
PaO_2_: Partial pressure of oxygen
PTB: Polypyrimidine tract-binding protein
SNP: Single nucleotide polymorphism
TDP-43: HIV-1 TAR DNA binding protein
TIA-1: T-cell intracellular antigen 1
