# Supplementary material for: Association of polymorphisms in genes of factors involved in regulation of splicing of cystic fibrosis transmembrane conductance regulator mRNA with acute respiratory distress syndrome in children with pneumonia
Source: Crit Care. 2016 Sep 5;20(1):281. doi: 10.1186/s13054-016-1454-7 (PMC5011993; doi:10.1186/s13054-016-1454-7)
Supplement: Additional file 1: Table S1. — Table of minor alleles and minor allele frequencies of genotyped variants. A table of the minor alleles and minor allele frequencies of genotyped variants in African American and Non-Hispanic Caucasian children with community acquired pneumonia. (DOCX 39 kb) [file 13054_2016_1454_MOESM1_ESM.docx]

| **Table 1S. Minor allele and minor allele frequencies of genotyped variants** | | | | | | | |
| --- | --- | --- | --- | --- | --- | --- | --- |
|  |  | African Americans | | | Non-Hispanic Caucasians | | |
| GENE | PROTEIN | SNPs | MA | MAF | SNPs | MA | MAF |
| *PTBP1* | PTB1 | rs351974 | A | 0.40 | rs351974 | C | 0.26 |
|  |  | rs351981 | C | 0.32 | rs351981 | C | 0.06 |
|  |  | rs10420953 | T | 0.06 | rs10420953 | T | 0.08 |
|  |  | rs736926 | T | 0.12 | rs736926 | T | 0.13 |
|  |  | rs4986234 | T | 0.03 | rs4986234 | T | 0.06 |
|  |  | rs3786992 | A | 0.05 | ND | - | - |
|  |  | ND | - | - | rs123698 | G | 0.38 |
| *SFRS1* | SR2/ASF | rs2233909 | G | 0.03 | ND | - | - |
|  |  | rs2233908 | G | 0.45 | ND | - | - |
|  |  | rs2233906 | C | 0.45 | ND | - | - |
|  |  | rs2233905 | T | 0.22 | ND | - | - |
|  |  | rs8819 | C | 0.49 | rs8819 | T | 0.17 |
|  |  | ND | - | - | rs2233911 | G | 0.35 |
|  |  | ND | - | - | rs16942573 | C | 0.06 |
| *SFRS5* | SRp40 | rs3104 | G | 0.42 | rs3104 | A | 0.26 |
|  |  | rs8014019 | A | 0.29 | ND | - | - |
|  |  | rs8013598 | C | 0.37 | ND | - | - |
|  |  | rs10144038 | C | 0.24 | ND | - | - |
|  |  | rs4899313 | A | 0.08 | rs4899313 | A | 0.09 |
|  |  | ND | - | - | rs8019166 | T | 0.15 |
|  |  | ND | - | - | rs11626887 | A | 0.02 |
| *TARDBP* | TDP-43 | rs12744501 | T | 0.20 | rs12744501 | C | 0.34 |
|  |  | rs7540903 | G | 0.16 | ND | - | - |
|  |  | rs2273348 | G | 0.24 | ND | - | - |
|  |  | rs1033638 | G | 0.38 | ND | - | - |
|  |  | rs7543605 | G | 0.01 | ND | - | - |
|  |  | rs12085877 | T | 0.11 | ND | - | - |
|  |  | rs3765896 | G | 0.18 | rs3765896 | G | 0.14 |
|  |  | rs1782455 | A | 0.33 | ND | - | - |
|  |  | rs3765897 | G | 0.08 | ND | - | - |
|  |  | ND | - | - | rs968545 | T | 0.05 |
|  |  | ND | - | - | rs12404672 | T | 0.05 |
|  |  | rs9430175 | C | 0.11 | ND | - | - |
| *TIA1* | TIA-1 | rs2289920 | C | 0.11 | rs2289920 | C | 0.06 |
|  |  | rs2166451 | C | 0.48 | rs2166451 | C | 0.33 |
|  |  | rs2592178 | A | 0.17 | ND | - | - |
|  |  | rs10175751 | T | 0.01 | ND | - | - |
|  |  | rs17005412 | G | 0.02 | ND | - | - |
|  |  | rs2706769 | C | 0.17 | ND | - | - |
|  |  | rs964392 | A | 0.17 | ND | - | - |
|  |  | rs13402990 | T | 0.03 | ND | - | - |
|  |  | rs11949 | G | 0.02 | ND | - | - |
|  |  | rs13024392 | A | 0.15 | ND | - | - |
|  |  | rs2921711 | C | 0.14 | ND | - | - |
|  |  | rs13411462 | A | 0.11 | ND | - | - |
|  |  | ND | - | - | rs2592177 | T | 0.30 |
| *U2AF2* | U2AF^65^ | rs7247677 | C | 0.24 | rs7247677 | T | 0.19 |
|  |  | rs537728 | G | 0.15 | ND | - | - |
|  |  | rs310445 | C | 0.22 | rs310445 | C | 0.05 |
|  |  | rs637788 | G | 0.11 | ND | - | - |
|  |  | rs540607 | C | 0.48 | rs540607 | T | 0.32 |
|  |  | rs2287791 | C | 0.30 | ND | - | - |
|  |  | rs10420401 | C | 0.16 | rs10420401 | C | 0.03 |
|  |  | rs507498 | A | 0.08 | ND | - | - |
|  |  | rs310442 | G | 0.19 | rs310442 | G | 0.02 |
|  |  | rs617073 | C | 0.33 | rs617073 | C | 0.09 |
|  |  | ND | - | - | rs2112799 | T | 0.17 |
| *CELF2* | CELF2 | rs17454839 | C | 0.17 | rs17454839 | C | 0.12 |
|  |  | rs1291833 | A | 0.11 | rs1291833 | A | 0.43 |
|  |  | rs3814634 | G | 0.38 | rs3814634 | T | 0.22 |
|  |  | rs17149511 | G | 0.20 | rs17149511 | G | 0.11 |
|  |  | rs6602473 | C | 0.24 | rs6602473 | C | 0.26 |
|  |  | rs10905928 | A | 0.11 | rs10905928 | A | 0.24 |
|  |  | rs2209285 | T | 0.39 | rs2209285 | T | 0.26 |
|  |  | rs17447321 | G | 0.05 | rs17447321 | G | 0.06 |
|  |  | rs1750729 | G | 0.26 | rs1750729 | G | 0.14 |
|  |  | rs2765980 | C | 0.06 | ND | - | - |
|  |  | rs606139 | A | 0.29 | rs606139 | G | 0.47 |
|  |  | rs201113 | T | 0.30 | rs201113 | C | 0.12 |
|  |  | rs17149653 | C | 0.21 | rs17149653 | C | 0.13 |
|  |  | rs2277212 | A | 0.25 | rs2277212 | A | 0.23 |
|  |  | rs11256967 | G | 0.27 | rs11256967 | G | 0.30 |
|  |  | rs1016540 | G | 0.14 | ND | - | - |
|  |  | rs7068124 | T | 0.31 | ND | - | - |
|  |  | rs4316428 | C | 0.08 | ND | - | - |

ND, not done because the variant was either monomorphic or was not a LD-tag SNP in the

indicated population.
